# Artificial intelligence and machine learning applications for the imaging of bone and soft tissue tumors

**DOI:** 10.3389/fradi.2024.1332535

**Published:** 2024-09-05

**Authors:** Paniz Sabeghi, Ketki K. Kinkar, Gloria del Rosario Castaneda, Liesl S. Eibschutz, Brandon K. K. Fields, Bino A. Varghese, Dakshesh B. Patel, Ali Gholamrezanezhad

**Affiliations:** ^1^ Department of Radiology, Keck School of Medicine, University of Southern California, Los Angeles, CA, United States; ^2^ Viterbi School of Engineering, University of Southern California, Los Angeles, CA, United States; ^3^ Keck School of Medicine, University of Southern California, Los Angeles, CA, United States; ^4^ Department of Radiology & Biomedical Imaging, University of California, San Francisco, San Francisco, CA, United States

**Keywords:** artificial intelligence, machine learning, deep learning, musculoskeletal, sarcoma

## Abstract

Recent advancements in artificial intelligence (AI) and machine learning offer numerous opportunities in musculoskeletal radiology to potentially bolster diagnostic accuracy, workflow efficiency, and predictive modeling. AI tools have the capability to assist radiologists in many tasks ranging from image segmentation, lesion detection, and more. In bone and soft tissue tumor imaging, radiomics and deep learning show promise for malignancy stratification, grading, prognostication, and treatment planning. However, challenges such as standardization, data integration, and ethical concerns regarding patient data need to be addressed ahead of clinical translation. In the realm of musculoskeletal oncology, AI also faces obstacles in robust algorithm development due to limited disease incidence. While many initiatives aim to develop multitasking AI systems, multidisciplinary collaboration is crucial for successful AI integration into clinical practice. Robust approaches addressing challenges and embodying ethical practices are warranted to fully realize AI's potential for enhancing diagnostic accuracy and advancing patient care.

## Key points

• Deep learning models have been developed for diagnosing MSK tumors and show potential to achieve diagnostic efficacy comparable to radiologists in limited classification tasks.
• AI algorithms can address issues related to variance in acquisition parameters and noise between MR scans using techniques such as edge-preserving denoising and intensity standardization.
• Multitasking AI systems that can efficiently perform multiple segmentation and analytical tasks at once hold promise for potentially useful prospective implementations in clinical practice.

## Introduction

Developments in artificial intelligence (AI) and machine learning (ML) have advanced the field of medicine and offer new and powerful digital tools to facilitate the next transformation in musculoskeletal (MSK) radiology. While it is important to acknowledge that these AI applications are still mainly in the experimental phase and need to be validated ahead of being fully integrated into standard clinical workflows, it is worth noting that they hold significant promise. In addition to streamlining radiology processes and enhancing the detection of abnormalities, AI techniques show potential for applications including predicting progression of malignancy and providing prognostic information (1–6). However, these potential advantages are not without some inherent biases and drawbacks, and radiologists must be aware of these pitfalls to allow for optimal implementation of AI tools in clinical practice (7, 8). AI may one day also enhance workflow productivity by automating repetitive processes, allowing radiologists to focus on image interpretation and clinical communication. Quality control may also come to be bolstered through enhanced automated detection of image artifacts and overall scan degradation. Finally, predictive analytics can help tailor interventions and allow for personalized modifications (9).

This review discusses key concepts and potential pitfalls of AI and ML in MSK radiology and how they can potentially be applied for diagnosis and treatment of soft tissue and bone tumors.

## Artificial intelligence and machine learning in medicine

AI generally refers to computer systems that simulate or mimic human intelligence (10). Beyond imaging interpretation in radiology, AI may also have a wide range of applications ranging from augmented structured reporting and clinical support systems to radiomics-based predictive implementations (11, 12).

ML defines a field of AI in which computers learn by analyzing large amounts of aggregated data and improve algorithms by iterative exposure and performance evaluation (11–14). ML subtypes can be categorized as supervised, unsupervised, reinforced, and semi-supervised. *Supervised* learning occurs via supplied output, while *unsupervised* learning develops from pattern recognition in input data without specific feedback. *Reinforcement* learning uses punishments or rewards as decision reinforcements. *Semi-supervised* learning involves fewer explicit outputs validated against a ground truth label (11).

Deep learning (DL), a subset of ML, is a multilayered approach which leverages hierarchical arrangement of multiple algorithms. Some of the more common applications of DL in MSK radiology include detection of spinal pathology, meniscal tears, fractures, and osteoarthritis (11, 15).

## Musculoskeletal radiology

MSK radiology employs a variety of imaging modalities to diagnose and assess disorders involving joints, muscles, soft tissues, and osseous structures. Imaging also plays a key role in initial assessment and treatment response characterization in bone tumors and soft-tissue sarcomas (STS) (16).

### Soft tissue tumors

Imaging techniques remain a pivotal component of the diagnosis of benign and malignant soft tissue lesions. While in many cases, specific clinical and imaging features may aid in narrowing down the differential diagnoses, definitive diagnosis is often made by tissue sampling and histopathologic interpretation (17). Though malignant tumors tend to be larger, some small soft tissue masses account for a significant portion of soft tissue malignancies. These smaller masses are more likely to be missed or be under-resected at surgery (18).

Lipomas and their malignant counterparts liposarcomas are among the soft tissue masses originating from adipose tissue (19). However, while well-differentiated liposarcomas (WDLs) and atypical lipomatous tumors (ALTs) can appear similar to intramuscular lipomas on imaging, the distinction holds significant implication for prognosis and treatment (20, 21). Specifically, treatment for higher-grade liposarcomas may require wide local excision with or without neoadjuvant or adjuvant chemotherapy and/or radiotherapy (19, 20). Similarly, while benign lipomas may in many instances be amenable to clinical observation or marginal resection, ALTs/WDLs may also require wide excision and subsequent imaging surveillance (22, 23).

Even though histology remains the gold standard for diagnosis, certain imaging modalities, mainly contrast enhanced MRI, may help to narrow the differential considerations ahead of tissue sampling. Nevertheless, traditional imaging modalities do possess inherent acknowledged limitations in reliably differentiating between benign and malignant soft tissue tumors (19, 22, 24).

### Bone tumors

Bone tumors can be classified into two main categories: primary tumors and secondary (metastatic) tumors. Malignant primary bone tumors arise from osseous tissues though have the potential to metastasize to other remote sites in the body (25).

Most benign bone tumors are chondrogenic in nature and are often found to be enchondromas or osteochondromas. Intermediate bone tumors such as giant cell tumors of bone may be at risk for malignant transformation. Chondrogenic and osteogenic tumors are among the most common primary bone malignancies (26).

Diagnosing bone tumors combines several approaches which consider clinical factors, histological sampling, and imaging (27). Radiography is often the initial imaging modality employed due to its ability to localize lesions and provide rapid holistic assessment of patterns of bony destruction, margins (zones of transition), and/or presence of periosteal reaction. These destructive patterns may provide insight into lesional biological activity and aggressiveness (2, 28).

MRI is the preferred method for local evaluation and staging due to its superiority in delineating associated soft-tissue components and detecting invasion into surrounding structures (29). Fluorine-18 fluorodeoxyglucose-PET scans can evaluate tumor metabolic activity, which often correlates with aggressiveness (27). PET/CT and PET/MRI are among the most sensitive and specific modalities for evaluating skeletal metastatic disease (30).

## Artificial intelligence and machine learning in musculoskeletal radiology

Various models have suggested that DL can, in relatively narrow use cases, achieve relatively similar diagnostic performance in comparison to human interpreting radiologists (31, 32). However, relative to other organ systems, the MSK system poses unique challenges for developing AI applications. The complex biomechanical interplay of the various anatomical structures makes it challenging for AI researchers to develop robust algorithms amidst the many possible scan angles and positional variations. Additionally, variability in acquisition parameters, image noise, and the field strength often necessitates complex preprocessing to improve and standardize image quality prior to AI operations (15).

Keles et al. (15) emphasize the importance of “clean data” for AI algorithms and discuss the need for preprocessing techniques. In the case of MRI, there are three main categories of challenges in medical images that need to be addressed with preprocessing, namely image nonstandardness, noise, and artifacts. The bias field artifact, also known as inhomogeneity, affects the quantitative intensity values of pixels and can in turn affect segmentation performance. In their preliminary studies, Keles et al. (15) applied generalized-scale post-processing to correct field inhomogeneities arising from the RF coil. Edge-preserving denoising was used to smooth images and thereby reduce image noise. To tackle signal intensity variations between MRI acquisitions, the authors applied an intensity standardization algorithm.

Segmentation of muscle, fat, and other regions of interest using automated techniques can be difficult due to overlapping intensity values of various tissues (15). LaLonde et al. present a DL algorithm called SegCaps, which was introduced for biomedical image segmentation tasks. The SegCaps algorithm leverages “deconvolutional capsules” in a design which purportedly requires fewer than 5% of the parameters necessary to execute the popular *U-Net* architecture (33).

Zhao et al. (34) developed three DL models utilizing ce-MRI to assist in the diagnosis of MSK neoplasms. This study's findings suggested that knowledge of the DL classifiers' predicted probability of malignancy led to increased sensitivity of imaging interpretation without significantly affecting specificity for providers of varying years of experience and training across oncology, MSK radiology, and orthopedic surgery (34).

DL may not only prove helpful in interpretation tasks but may also come to play a role in image reconstruction. Wessling et al. (35) employed a DL algorithm known as an “unrolled variational network”, which leverages an iterative parallel imaging reconstruction architecture to accelerate sequence acquisition time. Their results suggested that DL-based reconstruction both improved image quality and led to reductions in acquisition times of up to 52%–59% as compared to conventional scanning parameters (35).

## Artificial intelligence in imaging of soft tissue tumors

Regarding distinguishing lipomas from ALTs/WDLs in lipomatous soft tissue tumors, Leporq et al. (19) developed an MRI-based radiomics approach using fat-suppressed contrast-enhanced T1-weighted sequences and found that their classification models were able to distinguish between benign and malignant lesions. In their study, radiomics features were extracted from 2D, manually contoured tumor masks and subsequently used for machine learning. Their findings suggested that size features were most highly predictive of malignancy while intensity distribution features held the least predictive utility. However, they also found that shape features were most subject to interobserver variability.

In a recent study, Sudjai et al. (20) developed a machine-learning approach to differentiate between ALTs/WDLs and intramuscular lipomas based on radiomics features and the distance between tumor and bone on non-contrast T1-weighted MR images. The model achieved high accuracy in separating intramuscular lipomas from ALT/WDL, with an area under the curve (AUC) of 0.88. The model's performance was comparable to that of two MSK radiologists with 22 and 7 years of experience, respectively. Texture, shape, and histogram-based features were identified as most important in determining the model's predicted probability of malignancy.

Cay et al. (36) similarly found that a radiomics-based support vector machine algorithm was predictive of malignancy in lipomatous masses, with a reported sensitivity of 96.8% and a specificity of 93.72% for the machine learning approach. Regarding individual feature performance, gray-level run length matrix (GLRLM) based Run Length Non-Uniformity (RNLU) demonstrated the best performance, with an area under the curve (AUC) of 0.902 (36).

Fradet et al. (22) evaluated the relative performance of MRI radiomics with ML analysis with and without batch correction and DL models in predicting malignancy in lipomatous neoplasms. The authors performed a radiomics analysis on post-contrast fat-suppressed T1-weighted sequences with manual 3D segmentations. Best numerical results were seen with models trained on batch-corrected radiomics data (AUC of 0.80 vs. AUC of 0.70 in the external validation cohort for gradient boosting trained on radiomics data with and without batch correction, respectively). The Random Forest and Gradient Boosting models also notably outperformed the ResNet50 DL model in external validation, the latter of which only reached an AUC of 0.64.

Wang et al. (37) developed an ML radiomics-based nomogram for detecting malignancy in unknown soft tissue masses. The nomogram combined features of tumor margin, size, and capsule, along with a calculated radiomics score, yielding AUC values of 0.96 and 0.88 in validation testing.

Similarly, Fields et al. (24) reported that predictive models developed from radiomics data using machine learning-augmented approaches demonstrated effective discriminative capabilities in correctly categorizing benign and malignant lesions on preoperative MRI scans. Models built on unfiltered radiomics datasets yielded AUC values of 0.77 for Real Adaptive Boosting and 0.72 for Random Forest, respectively. Models limited to metrics derived only from T2 fat-saturated and Short-Tau Inversion Recovery sequences yielded similar performances, with AUCs of 0.73 for Real Adaptive Boosting and 0.75 for Random Forest. These results suggest that radiomics-based models based on restricted subsets of sequences may still maintain clinical relevance, which can help limit complexity and shorten analytical processing steps in future prospective implementations of machine learning-augmented workflows.

Navarro et al. (38) similarly developed DL models to stratify between high-grade and low-grade soft tissue tumors based on pre-treatment T2-weighted fat-saturated and contrast-enhanced T1-weighted fat-saturated MRIs. Following manual segmentation, separate DL models based on a pre-trained DenseNet-161 architecture were developed for each cohort of MRI sequences, which achieved AUCs of 0.76 and 0.75 for T2-weighted fat-saturated and contrast enhanced T1-weighted fat-saturated images, respectively. The DL models notably outperformed comparator regression models based on clinical features, tumor volume, and combined tumor volume and clinical features, respectively (38).

Multi-parametric MRI is the modality of choice for evaluation of treatment response in STS. However, the highly heterogeneous nature of these changes and varying degrees of tumor cellularity often confound evaluations and can contribute to clinical uncertainty (39, 40). In a cohort study by Blackledge et al., the authors suggested that ML can aid in evaluation of tumor response to radiotherapy (41). The authors evaluated the utility of eight different machine-learning approaches in differentiating between five distinct intratumoral tissue classes. Naïve-Bayes in combination with a Markov Random Field denoising algorithm was able to quantify changes in tumor sub-regions in a limited pre- and post-treatment cohort of 8 patients. These results suggest an ability for machine-learning techniques to assess underlying changes in tumor composition even in situations when overall changes in size in response to treatment may not be overtly evident.

Despite limitations in operator dependence, ultrasound may serve as a useful adjunctive modality for evaluation of soft-tissue masses. In a study by Wang et al. (42), the authors trained a convolutional neural network (CNN) to differentiate benign and malignant soft tissue lesions on routine clinical ultrasound, yielding an AUC of 0.91 and an accuracy of 79% on the test set. Sensitivity (90%) and specificity (74%) were not significantly different when compared to the performance of two interpreting MSK radiologists. Another CNN model was trained to distinguish between three different benign masses, namely benign nerve sheath tumors, vascular malformations, and lipomas. For the classification of lipomas, precision and recall of the model were 78% and 93%, respectively; for the classification of benign nerve sheath tumors, precision and recall were 71% and 42%, respectively; and for the classification of vascular malformations, precision and recall were 60% and 64%, respectively (42).

## Artificial intelligence in imaging of bone tumors

Several studies suggest a moderate to high accuracy for AI-based predictive models in differentiating between benign, intermediate and malignant tumors (1, 2, 32). Gitto et al. (43) examined how manual segmentation variability impacted the replicability of texture analysis on CT and MRI scans of cartilaginous bone tumors. The authors conducted 2D and 3D manual segmentations on CT and MRI scans, then implemented marginal shrinkage to assess impact on feature reproducibility. Overall, contour-focused segmentation yielded higher rates of stable radiomics features for 3D (80%) as compared to 2D (75%) regions-of-interest for CT and MRI. In comparison, marginal erosion performed more poorly for 3D features (*p* < 0.001) though was not statistically significant for 2D features (*p* = 0.343) (43).

He et al. (44) utilized DL on MRI images to predict the post-curettage local recurrence of giant cell tumor of bone based on preoperative MRI examinations. The results of CNN and CNN regression models were compared against the performance of four radiologists. The authors reported 75.5% accuracy and 85.7% sensitivity for the CNN model, and 78.6% accuracy and 87.5% sensitivity for the CNN regression model. This is in comparison to 64.3% accuracy and 58.3% sensitivity for the average performance of the radiologists (44).

## Challenges and future directions

Despite promising trends of applying AI and ML in MSK oncologic radiology, there exist several challenges and limitations to implementation. While there remains great interest in prospective implementations, achieving diagnostic accuracy is crucial. Practicing radiologists should strive to gain a thorough understanding of the use cases and associated challenges of AI implementation in prospective clinical workflows so as to maximize future applications in daily practice (14, 15).

Variations in implementation of AI applications is another major hurdle. Neural networks, which strive to replicate human cognition, require prolonged and frequently convoluted training and refinement stages. Furthermore, differences in implementation schemes across sites and institutions can significantly affect performance (11, 45, 46). Standardization of workflows will serve to promote generalizability and repeatability, which will increase diagnostic confidence in prospective applications (45).

Additionally, using such large amounts of patient data raise unique considerations with respect to data use ethics (47). In order to address these concerns, federated learning offers a unique approach in allowing for local training of a centrally-maintained AI model across many participating sites, thereby obviating the need for centralized data repositories (48).

DL techniques may one day aid in supporting clinical decision-making and automating certain lower-level tasks, allowing radiologists to focus greater attention on higher level interpretive tasks. Furthemore, radiomics and ML based classifiers working alongside other -omics may synergistically work to advance many AI modalities and more holistically capture unique aspects of the patient experience (10).
